# LIMK1/2 are required for actin filament and cell junction assembly in porcine embryos developing *in vitro*

**DOI:** 10.5713/ajas.19.0744

**Published:** 2020-01-13

**Authors:** Jeongwoo Kwon, Min-Jung Seong, Xuanjing Piao, Yu-Jin Jo, Nam-Hyung Kim

**Affiliations:** 1 Department of Animal Sciences, Chungbuk Natonal University, Cheongju 28864, Korea; 2 Primate Resources Center (PRC), Korea Research Institute of Bioscience and Biotechnology (KRIBB), Jeongeup 56216, Korea; 3 School of Biotechnology and Healthcare, Wuyi University, Jiangmen 529020, China

**Keywords:** Pig, Early Embryo, LIM Kinases (LIMK), Actin, Cell Junction Protein, Cell Division

## Abstract

**Objective:**

This study was conducted to investigate the roles of LIM kinases (LIMK1 and LIMK2) during porcine early embryo development. We checked the mRNA expression patterns and localization of LIMK1/2 to evaluate their characterization. We further explored the function of LIMK1/2 in developmental competence and their relationship between actin assembly and cell junction integrity, specifically during the first cleavage and compaction.

**Methods:**

Pig ovaries were transferred from a local slaughterhouse within 1 h and cumulus oocyte complexes (COCs) were collected. COCs were matured in *in vitro* maturation medium in a CO2 incubator. Metaphase II oocytes were activated using an Electro Cell Manipulator 2001 and microinjected to insert LIMK1/2 dsRNA into the cytoplasm. To confirm the roles of LIMK1/2 during compaction and subsequent blastocyst formation, we employed a LIMK inhibitor (LIMKi3).

**Results:**

LIMK1/2 was localized in cytoplasm in embryos and co-localized with actin in cell-to-cell boundaries after the morula stage. LIMK1/2 knockdown using LIMK1/2 dsRNA significantly decreased the cleavage rate, compared to the control group. Protein levels of E-cadherin and β-catenin, present in adherens junctions, were reduced at the cell-to-cell boundaries in the LIMK1/2 knockdown embryos. Embryos treated with LIMKi3 at the morula stage failed to undergo compaction and could not develop into blastocysts. Actin intensity at the cortical region was considerably reduced in LIMKi3-treated embryos. LIMKi3-induced decrease in cortical actin levels was attributed to the disruption of adherens junction and tight junction assembly. Phosphorylation of cofilin was also reduced in LIMKi3-treated embryos.

**Conclusion:**

The above results suggest that LIMK1/2 is crucial for cleavage and compaction through regulation of actin organization and cell junction assembly.

## INTRODUCTION

After fertilization by successful penetration of the sperm into the oocyte, a zygote undergoes the first cell division during the cleavage stage and subsequently undergoes continuous mitotic cell division. Subsequently, the blastomeres aggregate to form compacted morula and then develop into blastocysts. During early embryo development, various cell junction assemblies participate in cell division, cortical tension, cell polarity, and differentiation [1]. At the 2-cell stage, the adherens junction (AJ) become expressed at the apical regions [2]. While AJ proteins are expressed during the cleavage stage, tight junction (TJ) biogenesis occurs after the morula stage to aggregate the blastomeres [3]. E-cadherin, a transmembrane cell adhesion protein, functions in compaction and differentiation of the trophectoderm cell layer during early embryo development [4]. The TJ components, such as and coxsackievirus and adenovirus receptor (CXADR), are necessary for compaction, which ultimately leads to blastocyst formation [5]. These cell junction complexes are directly correlated with the cortical actin network and involved in the maintenance of morphogenesis [6].

Actin dynamics are regulated via polymerization and de-polymerization, by which F-actin is formed. This process is regulated by cofilin, which acts as the effector of Rho-ROCK-LIMK signaling [7]. Rho-associated protein kinase (ROCK) affects mammalian oocyte maturation by regulating cytoplasmic and cortical actin levels [8]. Loss of ROCK activity impairs actin dynamics, thereby causing failure of early embryo development [9]. The LIM kinases (LIMK) family includes two different kinases, LIMK1 and LIMK2, possessing serine/threonine and tyrosine activity, and these kinases phosphorylate the cofilin [10]. LIMK1/2 and p-cofilin directly prevent actin de-polymerization and maintain the actin filaments [11]. LIMK1/2, which act downstream of ROCK, are known to be required for microtubule assembly of the meiotic spindle and also required for accurate cell division and spindle positioning during mitosis [12]. LIMK1/2 regulate spindle organization and F-actin levels by phosphorylation of cofilin during mammalian oocyte maturation [13]. Inhibition of LIMK1/2 impairs cell division and disrupts actin dynamics during mouse early embryo development [14]. Although the mouse is a more beneficial experimental animal for developmental biology, different processes during early embryo development such as the timing of zygotic genome activation, the period between 1-cell to blastocyst formation, and gene expression patterns are more suitable for study in pigs. However, the effect of LIMK1/2 and the relationship between actin dynamics and cell junction integrity during cleavage and compaction remain unclear in pigs.

In the present study, we investigated the expression patterns and functional roles of LIMK1/2 during porcine embryo development using parthenotes. Parthenogenetic embryo development in mammals, especially in diploid embryos, is almost identical to *in vitro* fertilization (IVF) embryo developments, the polyspermy rate is very high, and they have low developmental rates compared to blastocysts in pig IVF embryos [15]. To elucidate the roles of LIMK1/2 during cleavage and the morula stage, respectively, we microinjected LIMK1/2 dsRNA to embryos in the 1-cell stage and treated morula stage embryos with LIMK inhibitor (LIMKi3). Our results showed that LIMK1/2 are crucial for cleavage and compaction, and that they act by regulating actin organization and cell junction assembly.

## MATERIALS AND METHODS

### Reagents

All reagents were purchased from Sigma-Aldrich (St. Louis, MO, USA), unless stated otherwise.

### Collection and *in vitro* maturation of porcine oocytes

All animal studies were performed in strict accordance with the institutional guideline and after prior approval from the Institutional Animal Care and Use Committee (IACUC) of the Chungbuk National University (CBNUA-1026-16-01). Prepubertal porcine ovaries were transported from a local abattoir (Farm Story Hannang, Cheongju, Korea) within 1 h of harvesting. Porcine cumulus-oocyte complexes (COCs) were recovered from follicles in porcine ovaries with diameters in the range of 3 to 6 mm. High-density cumulus oocytes were collected and washed thrice with TL-HEPES-PVA medium (HEPES medium supplemented with 0.01% polyvinyl alcohol). After washing, the collected COCs were cultured in *in vitro* maturation (IVM) medium for 44 h at 38.5°C in an atmosphere containing 5% CO_2_ at 100% humidity. The IVM medium (M-199; Invitrogen, Carlsbad, CA, USA) contained 20 μg/mL epidermal growth factor, 1 g/mL insulin, 75 g/mL kanamycin, 0.91 mM Na pyruvate, 0.57 mM L-cysteine, 10% (v/v) porcine follicular fluid, 0.5 μg/mL follicle stimulating hormone, and 0.5 μg/mL luteinizing hormone.

### Parthenogenetic activation and *in vitro* culture

For parthenogenetic activation, mature oocytes were denuded by gentle pipetting in 1 mg/mL hyaluronidase until all the cumulus cells around the oocyte were removed. Oocytes were then washed thrice in phosphate buffered saline-bovine serum albumin medium (PBS-BSA); Dulbecco’s phosphate-buffered saline (DPBS) added in 0.1% BSA and then activated using an Electro Cell Manipulator 2001 (BTX, Inc., San Diego, CA, USA). The electric pulse was stimulated at 1.1 kV/cm twice for 60 μs in 280 mM mannitol medium supplemented with 0.01 mM CaCl_2_ and 0.05 mM MgCl_2_. Activated oocytes were treated in PZM-5 medium containing 7.5 μg/mL cytochalasin B in an incubator for 3 h at 38.5°C. Embryos were washed thrice and cultured in PZM-5 for 144 h at 38.5°C in atmosphere containing 5% CO_2_.

### LIM kinase 1/2 knockdown by dsRNA injection

LIMK1- and 2-specific dsRNA primers were designed based on the sequences obtained from the National Center for Biotechnology Information database (XM_021086335.1) and are listed in Table 1. *In vitro* transcription of dsRNA was performed as previously described. Briefly, LIMK dsRNA was amplified using porcine blastocyst control cDNA and LIMK dsRNA primer. cDNA was purified using a gel extraction kit (Geneall Biotechnology, Seoul, Korea). Afterwards, *in vitro* dsRNA transcription was carried out at 37°C for 4 h using the MEGAscript T7 Transcription Kit (Ambion, Austin, TX, USA). DNase I-treated RNA was purified using mRNA filtered by RNeasy Mini Kit (Qiagen, Hilden, Germany). LIMK dsRNA was microinjected into porcine 1-cell embryos after parthenogenetic activation at 8 h using an Eppendorf Femto-Jet microinjector (Eppendorf, Hamburg, Germany) coupled with a Nikon TE2000-U inverted microscope (Nikon Corporation; Tokyo, Japan). After microinjection, the embryos were placed in PZM-5 medium and cultured in an incubator for 7 days.

### LIM kinase inhibitor (LIMKi3) treatment

To inhibit LIMK1/2 during early embryo development, morula, diluted in dimethyl sulfoxide (DMSO) were treated with LIMKi3 (Tocris Bioscience, Minneapolis, MN, USA). LIMKi3 was prepared at concentrations of 10, 50, 100, and 200 μM to evaluate the dose-dependent effect. Embryos were treated with LIMKi3 at 38.5°C in a humidified atmosphere containing 5% CO_2_. Embryos in the control group were cultured in DMSO at the same relative concentrations of solvent.

### Immunofluorescence analysis

Embryos were collected as previously described. Embryos were washed thrice in polyvinyl alcohol – phosphate buffer saline (PVA-PBS) and fixed with 3.7% paraformaldehyde in PBS for 30 min at room temperature. After washing thrice in PVA-PBS, embryos were permeabilized and blocked in DPBS containing 0.1% (v/v) Triton X-100 for 1 h at room temperature. Embryos were then incubated overnight at 4°C with the primary antibodies (Rabbit Anti-LIMK, Anti-P-cofilin, Anti-CXADR, Mouse Anti-ZO-1, Anti-E-cadherin, Anti-β-catenin) in the blocking solution, followed by incubation with Alexa Fluor 488- and 594-conjugated antibodies (Molecular Probes, Eugene, OR, USA) as secondary antibodies. Fluorescently labeled phalloidin (Sigma-Aldrich, USA) and Hoechst 33342 (10 mg/mL in PBS) were used to stain actin and nuclei, followed by several washes. Embryos were mounted onto glass slides and were examined using a confocal laser-scanning microscope (Zeiss LSM 710 META). The fluorescence intensity of LIMK1/2, p-cofilin, and phalloidin-stained actin were quantified using ImageJ software.

### Quantitative reverse transcriptase polymerase chain reaction

LIMK1/2 expression in porcine embryos was analyzed by quantitative reverse transcriptase polymerase chain reaction (PCR) using the ΔΔCT method. Total RNA was extracted from 25 embryos using a DynaBead mRNA Direct Kit (Thermo Fisher Scientific, Waltham, MA, USA). First-strand cDNA was generated using cDNA Synthesis Kit (LeGene, San Diego, CA, USA) and oligo(dT)20 primers. The PCR primers used to amplify LIMK1/2 are listed in Table 1. Real-time PCR was performed using the SuperGreen mix in a final reaction volume of 20 μL using reagents from a qPCR kit (WizPure qPCR Master). The following PCR conditions were used: 95°C for 10 min; and 39 cycles of 95°C for 10 s, 55°C or 65°C for 30 s, and final extension at 72°C for 10 min. Glyceraldehyde 3-phosphate dehydrogenase was used as an internal control for normalization in all analyses.

### Western blotting analysis

Control and LIMK1/2 dsRNA injected embryos were washed in PBS-PVA and placed at −80°C without medium before performing western blot. Embryos were contained in 1× sample sodium dodecyl sulfate buffer at 100°C for 10 min. Proteins were solubilized by electrophoresis and then separated on 10% sodium dodecyl sulfate–polyacrylamide gel electrophoresis gel and transferred to polyvinylidene fluoride membranes. Next, membranes were blocked in 1× triton-X–tris-buffer saline (TBS-T) with 5% skim milk for 1 h. Blocked membranes were contained in primary antibody (Rabbit Anti-LIMK; 1:1,000) placed at 4°C for overnight. Washed membranes were incubated for 1 h with HRP-conjugated anti-rabbit secondary antibody (Santa Cruz Biotechnology, Santa Cruz, CA, USA; 1:1,000). Signals were detected by UV transilluminator (UVITEC Cambridge, Cambridge, UK).

### Data analysis

All statistical analyses were performed using the Statistical Analysis System software (Statistical Analysis System. Inc. Cary, NC, USA). The experiments were performed with three biological replicates. The data were presented as mean± standard error of the mean. Statistical significance is defined when p values are less than 0.05.

## RESULTS

### Localization and expression patterns of LIMK1/2 during porcine embryo development

**Figure 1:**
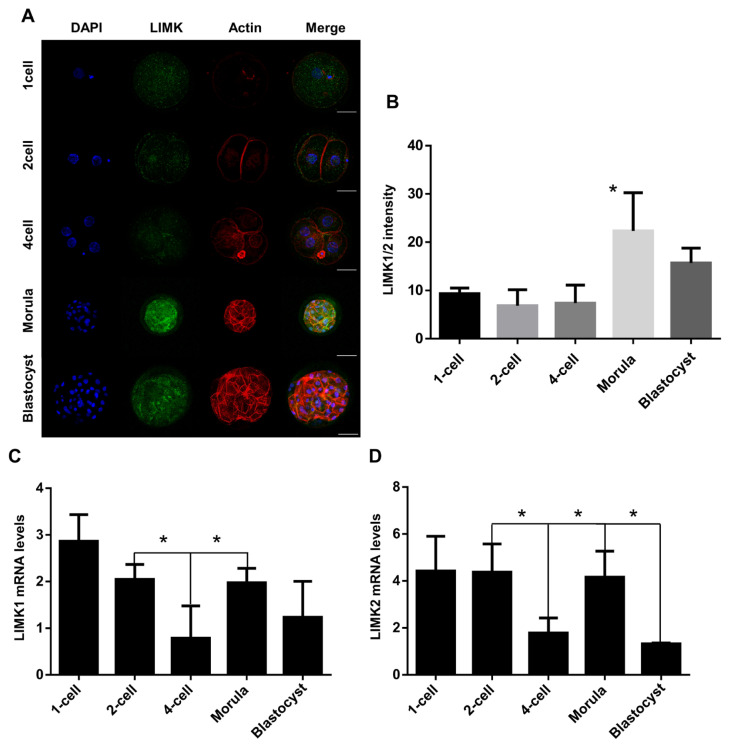
Localization and mRNA expression patterns of LIMK1/2 during porcine embryo development. (A) Immunocytochemistry of LIMK during early embryonic stage from the 1-cell to blastocyst stages. Embryos were collected at the 1-cell, 2-cell, 4-cell, morula, and blastocyst stages at 8, 36, 60, 96, and 144 h after parthenogenetic activation respectively. Blue, nucleus; green, LIMK; red, actin. Scale bar, 50 μm. (B) LIMK1/2 intensity during porcine early embryo development. Intensity of LIMK1/2 was calculated using ImageJ software (C) Relative mRNA levels of LIMK1 and LIMK2 during porcine early embryo development. LIMK, LIM kinases.

Immunofluorescence staining in each developmental stage was performed to determine LIMK1/2 localization. Quantitative real-time polymerase chain reaction (qRT-PCR) was performed to validate LIMK1/2 expression levels during porcine embryo development. Embryos were sampled at the 1-cell, 2-cell, 4-cell, morula, and blastocyst stages at 8, 36, 60, 96, and 144 h after activation, respectively. Results confirmed that LIMK1/2 were scattered randomly in the cytoplasm from the 1-cell to 4-cell stages (Figure 1A). From the morula stage, LIMK1/2 were located at the cell junction boundaries near actin. Additionally, LIMK1/2 intensity were increased after the morula stage (Figure 1B). The mRNA of LIMK1 and LIMK2 showed no significant differences in their expression levels between the 4-cell stage and the 1-cell stage, while they were found to be upregulated at the morula stage (Figure 1C, 1D). These results indicated that LIMK1/2 are expressed after the morula stage, and that they localized at the cell boundaries during early embryo development.

### Arrest of porcine early embryonic development by knockdown of LIMK1/2

**Figure 2:**
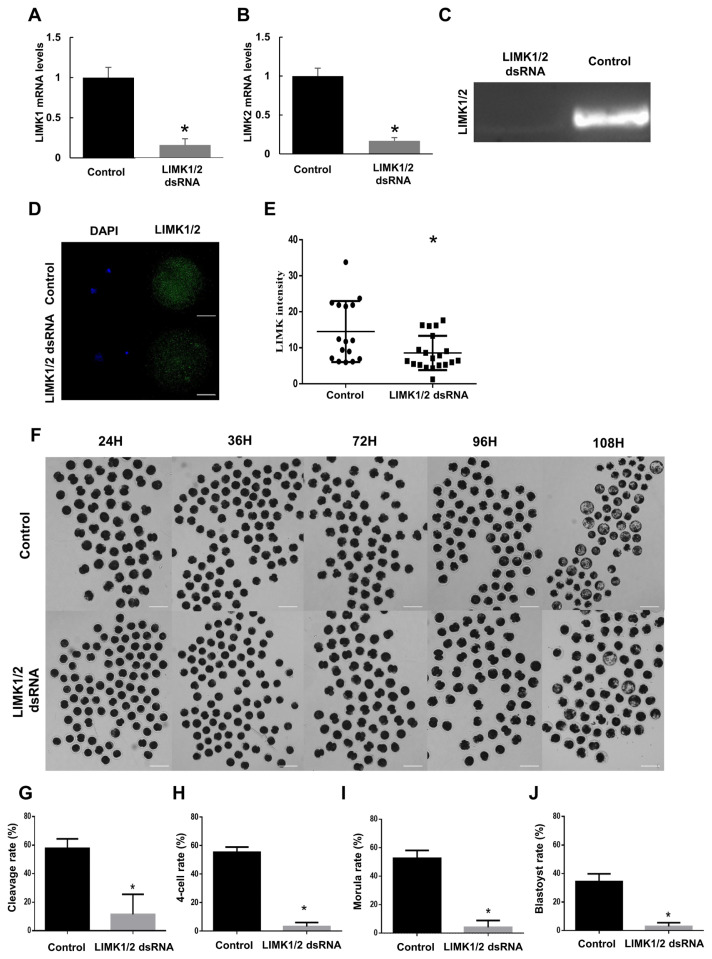
Effect of disrupting LIMK activity on embryo development. (A and B) Knockdown of LIMK1/2 using LIMK1 and 2 dsRNA microinjection into 1-cell embryos. mRNA levels were confirmed by qRT-PCR after 24 h of microinjection. (C) Protein levels of control and LIMK1/2 dsRNA injection groups by western blot. (D and E) Immunostaining of LIMK1/2 and intensity of LIMK1/2. Scale bars, 50 μm. (F) Developmental competence by knockdown of LIMK1/2. The control group was microinjected with eGFP dsRNA, while the knockdown group was microinjected with LIMK dsRNA. Scale bars, 200 μm. (G–J) Embryo developmental rates at the cleavage, 4-cell, morula and blastocyst stages after LIMK knockdown. Over 30 embryos was used for the qRT-PCR and immunostaining. Analysis of developmental rates was calculated for more than 50 embryos in each group. LIMK, LIM kinases; qRT-PCR, quantitative real-time polymerase chain reaction; eGFP, enhanced green fluorescent protein. * p<0.01.

To confirm the role of LIMK during porcine early embryo development, LIMK 1 and 2 dsRNA were microinjected to embryos in the 1-cell stage. LIMK 1 and 2 mRNA levels were downregulated in the LIMK1/2 knockdown group in comparison with the control group (Figure 2A, 2B). After LIMK1/2 knockdown, the intensity of the LIMK1/2 signals, and the LIMK1/2 protein were significantly lower than those in the control group (Figure 2C–2E). These results indicated that injection with LIMK1/2 dsRNA had high knockdown efficiency. The cleavage rate was significantly lower in the LIMK1/2 knockdown group than that in the control group (Figure 2F, 2G; control, 57.73±3.870 vs LIMK1/2 knockdown, 11.53± 8.087). In addition, the 4-cell (control, 55.22±2.118 vs LIMK1/2 knockdown, 5.314±1.433), morula (control, 49.88±1.598 vs LIMK1/2 knockdown, 4.125±2.764), and blastocyst rates (control, 34.41±2.424 vs LIMK1/2 knockdown, 2.949±1.299) were significantly lower in the knockdown group Figure 2H–2J). Results showed that most of the embryos in the LIMK1/2 knockdown group did not undergo cell division and arrested their development at the 1-cell stage.

### Disrupting LIMK activity causes an abnormal division and breakdown of adherens junction proteins

**Figure 3:**
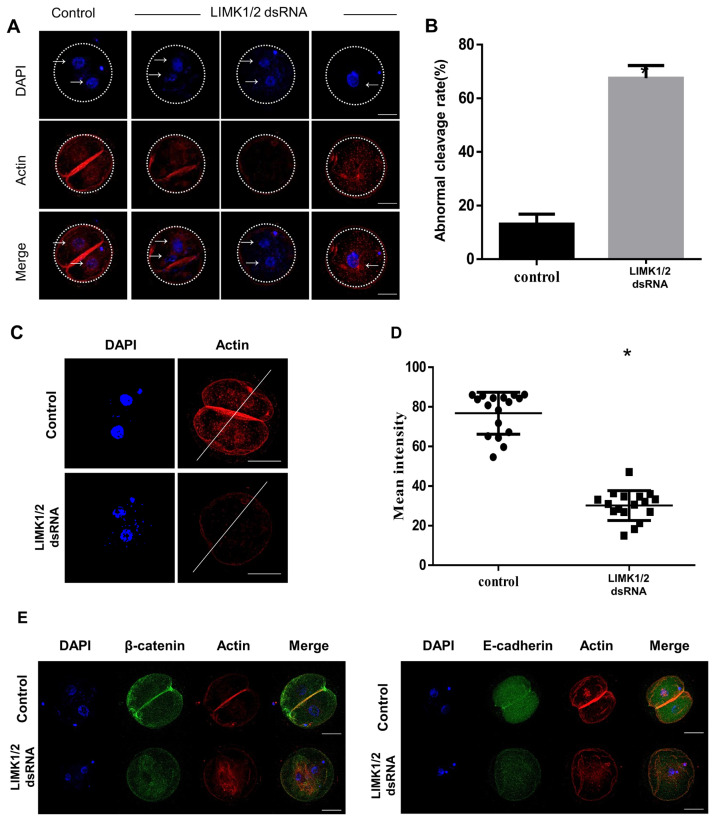
Abnormal cytokinesis following disrupted LIMK activity on actin and adherens junction proteins. (A and B) Abnormal cell division after LIMK1/2 knockdown. After 24 h of microinjection of LIMK1/2 dsRNA, embryos were stained with Actin-phalloidin to visualize actin (red), and Hoechst 33342 was used for DNA staining (blue). Type 1, asymmetric cell division and blastomeres have two nuclei; Type 2, 2 nuclei in 1 cell embryo; Type 3, 1-cell arrest. Abnormal cytokinesis in 2-cell stage included asymmetric cell division, no cell-to-cell membrane (without actin), 1-cell arrest and abnormal actin distribution. Arrows indicate the nucleus. (C and D) Reduction in actin intensity following LIMK1/2 knockdown. Actin fluorescence intensity was calculated by the while line. Mean actin intensity was decreased in LIMK1/2 knockdown embryos. Blue, nucleus; red, actin. Scale bars, 50 μm. (E) Disruption of adherens junction proteins in LIMK1/2 knockdown embryos. β-Catenin and E-cadherin were not detected in the cell-to-cell boundaries. Blue, nucleus; green, β-catenin or E-cadherin; red, actin. LIMK, LIM kinases. Scale bars, 50 μm. * p<0.01.

Previous research reported that cortical actin and AJ are crucial for first cleavage during early embryo development [16]. Therefore, we conducted immunofluorescence staining to evaluate the relationship between actin and junction proteins. LIMK1/2 knockdown embryos had asymmetric cleavage or cleavage of the nucleus alone and had failed cleavage formation, while most embryos in the control groups were successfully formed by symmetric division (Figure 3A). The rate of abnormal cleavage was significantly higher in LIMK1/2 knockdown embryos (Figure 3B, control, 13.12±2.144 vs LIMK1/2, 67.53±2.703). Furthermore, cortical and cytoplasmic actin intensities were significantly lower in the LIMK1/2 knockdown group (Figure 3C, 3D; control, 76.80±2.574 vs LIMK1/2, 30.19±1.827). LIMK1/2 dsRNA-injected embryos showed abnormal β-catenin and E-cadherin localization with actin in cytoplasm, while control embryos showed normal localization in the cell membrane (Figure 3E, 3F).

### Treatment with LIMKi3 interferes with blastocyst formation by disruption of cell junctions with actin

**Figure 4:**
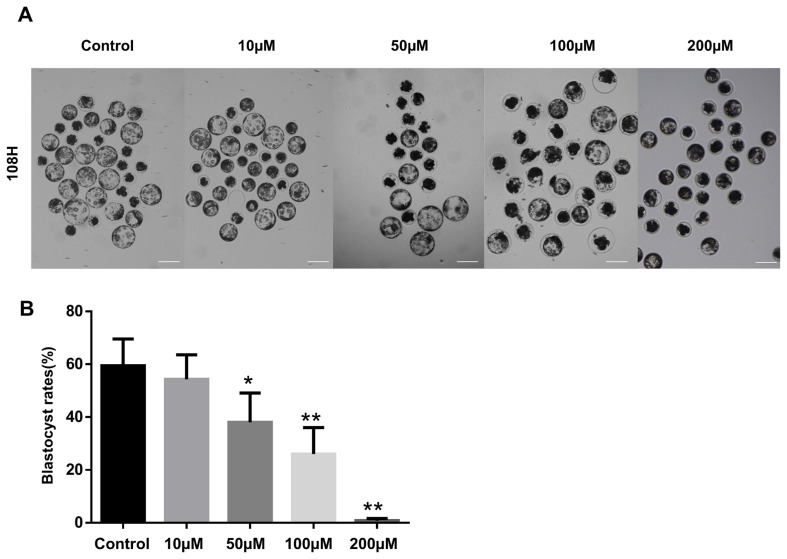
Decreasing of blastocyst rate by treatment of LIMKi3. (A and B) Effects of LIMKi3 during early embryo development. LIMKi3 was treated at the morula stage with different concentrations (0, 10, 50, 100, and 200 μM). The blastocyst formation rates reduced in adose-dependent manner. LIMK, LIM kinases; LIMKi3, LIMK inhibitor. Scale bars, 200 μm. * p<0.05, ** p<0.01.

**Figure 5:**
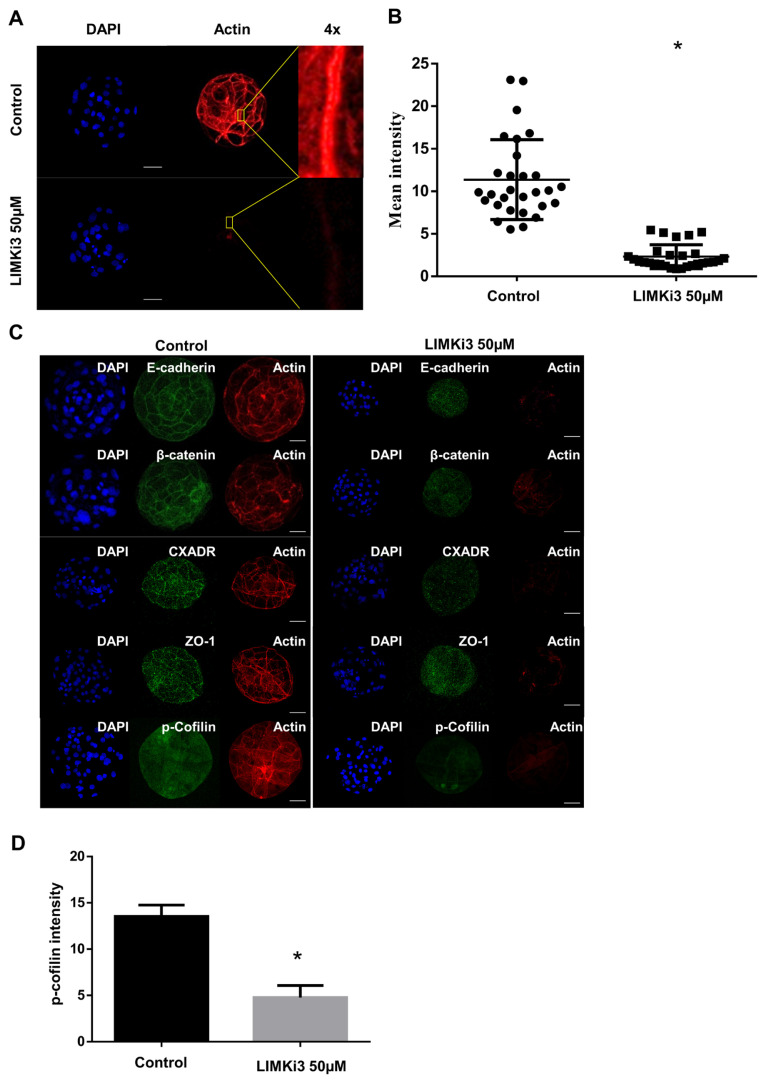
Effect of LIMK inhibition on embryos actin with cell junction. (A and B) Decreasing actin levels after LIMKi3 treatment at the morula stage. In the cell-to-cell boundaries, actin intensity was decreased in LIMKi3 treatment group compared with the control group. Blue, nucleus; red, actin. Scale bars, 50 μm. (C) Cell junction assembly breakdown in LIMKi3 treated embryos. After treatment of LIMKi3, AJ, and TJ proteins were decreased with actin and p-cofilin. Blue, nucleus; green, β-catenin, E-cadherin, ZO-1, CXADR or p-Cofilin; red, actin. (D) p-cofilin level after treatment of LIMKi3 in blastocyst stage. The intensity of p-cofilin decreased in LIMKi3 treated embryos. Intensity of p-cofilin was calculated using ImageJ software. LIMK, LIM kinases; LIMKi3, LIMK inhibitor. Scale bars, 50 μm.

We treated 1-cell embryos with LIMKi3 (LIMK1 and LIMK2 inhibitor). Treatment with 1 μM and 10 μM of LIMKi3 at 1-cell embryos showed no difference of cleavage rates. However, treatment 50 μM of LIMKi3 showed decreased cleavage rates and 1-cell embryos did not develop into 2-cell embryos. In samples treated with LIMKi3 at the morula stage, the 10 μM LIMKi3 group and the control group showed no significant differences in blastocyst formation rates, but embryos treated with 50, 100, and 200 μM LIMKi3 showed significantly lower blastocyst rates (Figure 4A, 4B). To verify the relationship between LIMK1/2 and cell junction integrity during compaction to blastocyst formation, we conducted the experiment by setting the concentration of the treatment group to 50 μM. The integrity of the association of tight and AJ proteins with actin was determined. LIMKi3-treated blastocysts showed significantly lower actin intensities than blastocysts in the control group at the cell junction areas (Figure 5A, 5B). In addition, both signals, corresponding to the AJ proteins β-catenin and E-cadherin, and the TJ proteins ZO-1 and CXADR, were disrupted in the LIMKi3 treatment group relative to the control group (Figure 5C). LIMKi3-treated embryos showed significantly reduced cofilin phosphorylation levels compared to embryos in the control group (Figure 5D).

## DISCUSSION

LIMK acts downstream of Rho-GTPase and is mainly distributed in actin synthesis sites, involved in the regulation of cytoskeletal dynamics and cell proliferation during embryo development [14]. Previous studies showed that another Rho-GTPase effector, ROCK, which is located in the nucleus and cytoplasm [9], is required for blastocyst cavity formation and normal ICM differentiation [17]. In pigs, ROCK 1 and ROCK 2 were detected in the cell junction regions and nucleus, respectively, and ROCK isoforms appeared to have distinct cellular functions that are dependent on their expression and localization [8]. Our results showed that LIMK1 and LIMK2 mRNA levels have similar expression patterns as ROCK1 mRNA levels during early embryo development, increasing after the morula stage [8]. The LIMK1/2 proteins were detected at the cytoplasm during the 2-cell stage and subsequently translocated to the apical cell regions after compaction, which was similarly observed for ROCK 2 proteins [8]. p-LIMK1/2 were localized in the cytoplasm and cortex during oocyte maturation, and they accumulated at the actin cap and polar body during metaphase I and II [18]. Inactivation of LIMK1/2 was found to decrease the cytoplasmic actin mesh, following abnormal meiotic cell division during oocyte maturation. Our results showed that LIMK1/2 knockdown embryos did not undergo normal cell division and showed abnormal actin distribution in the 2-cell stage. Therefore, our results demonstrated that similar to ROCK, LIMK1/2 has a functional role in cell division by affecting actin network during porcine cleavage.

Our results showed that LIMK1/2 knockdown in embryos lead to an abnormal cell division, following the decrease of cortical actin levels (Figures 2, 3). Actin is distributed in the cytoplasm and in the cortical area of embryos at cleavage furrow after fertilization and subsequent development [19]. Actin cytoskeleton structure is regulated by cofilin through ROCK/LIMK1/2 signaling [20]. AJ proteins directly bind F-actin at cortical areas and contribute to morphogenesis in epithelial cells [21]. E-cadherin and β-catenin are present at the 2-cell stage from residual maternal gene transcripts or proteins before zygotic genome activation [22]. These AJ proteins act in a coordinated manner and are required for morphogenesis and the growth of embryos [23]. In abnormal 2-cell embryos that are produced after LIMK1/2 knockdown, E-cadherin and β-catenin cannot be localized within the cell junction boundaries via actin dynamic disruption. Although we did not show the interaction between the intracellular actin network and AJ complex, the breakdown of actin and AJ proteins as LIMK1/2 knockdown suggested that LIMK1/2-mediated actin dynamics is crucial for cytokinesis and the maintenance of AJ assembly.

In this study, inhibition of LIMK1/2 interfered with blastocyst formation (Figure 4A, 4B) and decreased cortical actin levels after LIMKi3 treatment at the morula stage (Figure 5A, 5B). In addition, the results showed that LIMK1/2 downregulation caused overall disruption of actin and cell junction integrity because of the dysregulation of proteins, such as E-cadherin, β-catenin, ZO-1, and CXADR. At the apical regions in each blastomere, cell-to-cell junction assembly coordinates with actin dynamics in the compaction stage [24]. In various cell junction complexes, AJ and TJ-associated genes are expressed, and they play functional roles in cytokinesis and compaction in embryos [1,4,25]. Cell junction integrity is directly correlated with the formation of actin networks [23], which is regulated by the Rho family GTPases and their effectors [26]. Actin cytoskeleton dynamics are important for the maintenance of TJs by LIMK1/2-mediated cofilin phosphorylation and de-phosphorylation in human intestinal cells [27]. In our previous studies, cell junction regulatory proteins were shown to be crucial for compaction leading to blastocyst formation, and they act by regulating other AJ and TJ proteins that maintain integrity [5]. Moreover, ROCK activity regulates TJ and AJ assembly by altering the expression patterns of genes related to TJ and AJ in early embryo development [28]. Taken together, previous findings and our current results suggested that ROCK/LIMK1/2 signaling during early embryo development is not only involved in actin dynamics, but also in cell junction assembly.

Members of the ROCK family of proteins phosphorylate LIMK1/2, and activated LIMK1/2 assemble the actin network by inducing cofilin expression [20]. Cofilin is an actin-severing protein that belongs to the actin depolymerizing factor/cofilin family that promotes actin dynamics by de-polymerizing and severing preexisting filaments [29]. Phosphorylation of cofilin (p-cofilin) on the N-terminal serine-3 residue caused inactivation of cofilin, regulated by LIMK1/2 activity [16]. Downregulation of cofilin1 expression not only upregulated the E-cadherin expression, but it also accelerated compaction during early mouse development [30]. Our results showed that p-cofilin intensity was decreased after LIMKi3 treatment, following breakdown of AJ and TJ integrity. The above results demonstrated that LIMK/cofilin pathways are important for the maintenance of cell junctions during porcine early embryo development.

In summary, LIMK1/2 are expressed during early embryo development, and they co-localize with actin at the cell-to-cell junctions after the morula stage. Our results showed that knockdown of LIMK1/2 activity impairs the first cell division after parthenogenetic activation. Furthermore, we showed that inhibition of LIMK1/2 at the morula stage caused failure of blastocyst formation. Loss of LIMK1/2 activity following LIMKi3 treatment downregulated cytoplasmic and cortical actin levels and interfered with overall cell junction integrity. Moreover, breakdown of dynamic actin network and cell junction assembly was caused by the downregulation of the p-cofilin level. Therefore, the results indicated that LIMK1/2 plays a role in the cleavage and compaction by actin assembly and cell junction complex integrity in porcine early-stage embryos.

## Figures and Tables

**Table 1:** Primer sequences for dsRNA and qRT-PCR

Gene	GenBank accession No.	Sequence (5′– 3′)	Amplicon size (bp)
*LIMK1* dsRNA	XM_021086335.1	F: TAATACGACTCACTATAGGGAGACCACTGGACGAGATTGATCTGCTG	613
		R: TAATACGACTCACTATAGGGAGACCACAAGCTGACCCTCTGACTCCA	
*LIMK2* dsRNA	XM_005670864.3	F: TAATACGACTCACTATAGGGAGACCACATGCACATCAGTCCCAACAA	649
		R: TAATACGACTCACTATAGGGAGACCACGCAGGTTCAGCTTCTTGTCC	
*eGFP* dsRNA	NC_025025.1	F: ATTAATACGACTAACTATAGGGAGAATGGTGAGCAAGGGCGAG	651
		R: ATTAATACGACTCACTATAGGGAGAGCTCGTCCATGCCGAGAG	
*LIMK1* for qRT-PCR	XM_021086335.1	F: AACTGCCTGGTTCGAGAGAA	221
		R: ACGATGCCAAAGGAAAACAC	
*LIMK2* qRT-PCR	XM_005670864.3	F: GGGAGAAGTTTGTTCCCACA	196
		R: TGCAGGCTCACAGTATGGTC	
*GAPDH*	NM_001206359.1	F: GGAGAACGGGAAGCTTGTCA	224
		R: GGTTCACGCCCATCACAAAC	

F, forward; R, reverse.

qRT-PCR, quantitative real-time polymerase chain reaction; *LIMK*, LIM kinases; *eGFP*, enhanced green fluorescent protein; *GAPDH*, glyceraldehyde 3-phosphate dehydrogenase.

## References

[1] Hardy K, Warner A, Winston RM, Becker DL (1996). Expression of intercellular junctions during preimplantation development of the human embryo. Mol Hum Reprod.

[2] Fleming TP, Sheth B, Fesenko I (2001). Cell adhesion in the preimplantation mammalian embryo and its role in trophectoderm differentiation and blastocyst morphogenesis. Front Biosci.

[3] Sheth B, Moran B, Anderson JM, Fleming TP (2000). Post-translational control of occludin membrane assembly in mouse trophectoderm: a mechanism to regulate timing of tight junction biogenesis and blastocyst formation. Development.

[4] Ohsugi M, Larue L, Schwarz H, Kemler R (1997). Cell-junctional and cytoskeletal organization in mouse blastocysts lacking E-cadherin. Dev Biol.

[5] Kwon JW, Kim NH, Choi I (2016). CXADR is required for AJ and TJ assembly during porcine blastocyst formation. Reproduction.

[6] Heisenberg CP, Bellaiche Y (2013). Forces in tissue morphogenesis and patterning. Cell.

[7] Song X, Chen X, Yamaguchi H (2006). Initiation of cofilin activity in response to EGF is uncoupled from cofilin phosphorylation and dephosphorylation in carcinoma cells. J Cell Sci.

[8] Zhang JY, Dong HS, Oqani RK, Lin T, Kang JW, Jin DI (2014). Distinct roles of ROCK1 and ROCK2 during development of porcine preimplantation embryos. Reproduction.

[9] Duan X, Chen KL, Zhang Y, Cui XS, Kim NH, Sun SC (2014). ROCK inhibition prevents early mouse embryo development. Histochem Cell Biol.

[10] Scott RW, Olson MF (2007). LIM kinases: function, regulation and association with human disease. J Mol Med (Berl).

[11] Bamburg JR, Bernstein BW (2010). Roles of ADF/cofilin in actin polymerization and beyond. F1000 Biol Rep.

[12] Kaji N, Muramoto A, Mizuno K (2008). LIM kinase-mediated cofilin phosphorylation during mitosis is required for precise spindle positioning. J Biol Chem.

[13] Li X, Zhu Y, Cao Y (2016). LIM kinase activity is required for microtubule organising centre positioning in mouse oocyte meiosis. Reprod Fertil Dev.

[14] Duan X, Zhang HL, Wu LL (2018). Involvement of LIMK1/2 in actin assembly during mouse embryo development. Cell Cycle.

[15] Kharche SD, Birade HS (2013). Parthenogenesis and activation of mammalian oocytes for *in vitro* embryo production: a review. Adv Biosci Biotechnol.

[16] Tanaka K, Okubo Y, Abe H (2005). Involvement of slingshot in the Rho-mediated dephosphorylation of ADF/cofilin during *Xenopus* cleavage. Zoolog Sci.

[17] Laeno AM, Tamashiro DA, Alarcon VB (2013). Rho-associated kinase activity is required for proper morphogenesis of the inner cell mass in the mouse blastocyst. Biol Reprod.

[18] Jia RX, Duan X, Song SJ, Sun SC (2016). LIMK1/2 inhibitor LIMKi 3 suppresses porcine oocyte maturation. PeerJ.

[19] Gumus E, Bulut HE, Kaloglu C (2010). Cytoskeletal changes in oocytes and early embryos during *in vitro* fertilization process in mice. Anat Histol Embryol.

[20] Maekawa M, Ishizaki T, Boku S (1999). Signaling from rho to the actin cytoskeleton through protein kinases ROCK and LIM-kinase. Science.

[21] Nagafuchi A (2001). Molecular architecture of adherens junctions. Curr Opin Cell Biol.

[22] De Vries WN, Evsikov AV, Haac BE (2004). Maternal beta-catenin and E-cadherin in mouse development. Development.

[23] Stephenson RO, Yamanaka Y, Rossant J (2010). Disorganized epithelial polarity and excess trophectoderm cell fate in preimplantation embryos lacking E-cadherin. Development.

[24] Zenker J, White MD, Gasnier M (2018). Expanding actin rings zipper the mouse embryo for blastocyst formation. Cell.

[25] Sheth B, Fesenko I, Collins JE (1997). Tight junction assembly during mouse blastocyst formation is regulated by late expression of ZO-1 alpha+ isoform. Development.

[26] Braga VM, Machesky LM, Hall A, Hotchin NA (1997). The small GTPases Rho and Rac are required for the establishment of cadherin-dependent cell-cell contacts. J Cell Biol.

[27] Nagumo Y, Han J, Bellila A, Isoda H, Tanaka T (2008). Cofilin mediates tight-junction opening by redistributing actin and tight-junction proteins. Biochem Biophys Res Commun.

[28] Kwon J, Kim NH, Choi I (2016). ROCK activity regulates functional tight junction assembly during blastocyst formation in porcine parthenogenetic embryos. PeerJ.

[29] Ohashi K (2015). Roles of cofilin in development and its mechanisms of regulation. Dev Growth Differ.

[30] Ma M, Zhou L, Guo X (2009). Decreased cofilin1 expression is important for compaction during early mouse embryo development. Biochim Biophys Acta Mol Cell Res.

